# IMI2-PainCare-BioPain-RCT1: study protocol for a randomized, double-blind, placebo-controlled, crossover, multi-center trial in healthy subjects to investigate the effects of lacosamide, pregabalin, and tapentadol on biomarkers of pain processing observed by peripheral nerve excitability testing (NET)

**DOI:** 10.1186/s13063-022-06087-1

**Published:** 2022-02-19

**Authors:** Zahra Nochi, Hossein Pia, Petra Bloms-Funke, Irmgard Boesl, Ombretta Caspani, Sonya C. Chapman, Francesca Fardo, Bernd Genser, Marcus Goetz, Anna V. Kostenko, Caterina Leone, Thomas Li, André Mouraux, Bernhard Pelz, Esther Pogatzki-Zahn, Andreas Schilder, Erik Schnetter, Karin Schubart, Alexandre Stouffs, Irene Tracey, Iñaki F. Troconiz, Andrea Truini, Johannes Van Niel, Jose Miguel Vela, Katy Vincent, Jan Vollert, Vishvarani Wanigasekera, Matthias Wittayer, Hatice Tankisi, Nanna B. Finnerup, Keith G. Phillips, Rolf-Detlef Treede

**Affiliations:** 1 Danish Pain Research Center, Department of Clinical Medicine, Aarhus University, Aarhus, Denmark; 2 Translational Science & Intelligence, Grünenthal GmbH, Aachen, Germany; 3 Clinical Science Development, Grünenthal GmbH, Aachen, Germany; 4 Department of Neurophysiology, Mannheim Center for Translational Neurosciences (MCTN), University of Heidelberg, Mannheim, Germany; 5 Eli Lilly and Company, Arlington Square, Bracknell, UK; 6 Mannheim Institute of Public Health, Social & Preventive Medicine, University of Heidelberg, Heidelberg, Germany; 7 MRC Systems GmbH, Heidelberg, Germany; 8 Department of Human Neuroscience, Sapienza University, Rome, Italy; 9 Teva Branded Pharmaceutical Products R&D, Inc, West Chester, PA USA; 10 Institute of Neuroscience (IoNS), UCLouvain, Brussels, Belgium; 11 Department of Anaesthesiology, Intensive Care and Pain Medicine, University Hospital Münster, Münster, Germany; 12 University Computing Centre, University of Heidelberg, Heidelberg, Germany; 13 ConsulTech GmbH, Berlin, Germany; 14 Wellcome Centre for Integrative Neuroimaging, FMRIB Centre, Nuffield Department of Clinical Neurosciences, University of Oxford, John Radcliffe Hospital, Oxford, OX3 9DU UK; 15 Department of Pharmaceutical Technology and Chemistry, School of Pharmacy and Nutrition, University of Navarra, Pamplona, Spain; 16 Mature Products Development, Grünenthal GmbH, Aachen, Germany; 17 Welab Barcelona, Barcelona, Spain; 18 Nuffield Department of Women’s and Reproductive Health (NDWRH), University of Oxford, Oxford, UK; 19 Pain Research, Department of Surgery and Cancer, Imperial College London, London, UK; 20 Department of Clinical Medicine, Aarhus University and Department of Clinical Neurophysiology, Aarhus University Hospital, Aarhus, Denmark; 21 Neuroscience Next Generation Therapeutics, Eli Lilly and Company, Lilly Innovation Center, Cambridge, MA USA

**Keywords:** Pain, Analgesics, PK/PD, Nerve excitability testing, Threshold tracking, Biomarkers, Hyperalgesia, RCT, Healthy subjects, Ectopic impulse generation

## Abstract

**Background:**

Few new drugs have been developed for chronic pain. Drug development is challenged by uncertainty about whether the drug engages the human target sufficiently to have a meaningful pharmacodynamic effect. IMI2-PainCare-BioPain-RCT1 is one of four similarly designed studies that aim to link different functional biomarkers of drug effects on the nociceptive system that could serve to accelerate the future development of analgesics. This study focusses on biomarkers derived from nerve excitability testing (NET) using threshold tracking of the peripheral nervous system.

**Methods:**

This is a multisite single-dose, subject and assessor-blind, randomized, placebo-controlled, 4-period, 4-way crossover, pharmacodynamic (PD), and pharmacokinetic (PK) study in healthy subjects. Biomarkers derived from NET of large sensory and motor fibers and small sensory fibers using perception threshold tracking will be obtained before and three times after administration of three medications known to act on the nociceptive system (lacosamide, pregabalin, tapentadol) and placebo, given as a single oral dose with at least 1 week apart. Motor and sensory NET will be assessed on the right wrist in a non-sensitized normal condition while perception threshold tracking will be performed bilaterally on both non-sensitized and sensitized forearm skin. Cutaneous high-frequency electrical stimulation is used to induce hyperalgesia. Blood samples will be taken for pharmacokinetic purposes and pain ratings as well as predictive psychological traits will be collected. A sequentially rejective multiple testing approach will be used with overall alpha error of the primary analysis split across the two primary outcomes: strength-duration time constant (SDTC; a measure of passive membrane properties and nodal persistent Na^+^ conductance) of large sensory fibers and SDTC of large motor fibers comparing lacosamide and placebo. The key secondary endpoint is the SDTC measured in small sensory fibers. Remaining treatment arm effects on key NET outcomes and PK modelling are other prespecified secondary or exploratory analyses.

**Discussion:**

Measurements of NET using threshold tracking protocols are sensitive to membrane potential at the site of stimulation. Sets of useful indices of axonal excitability collectively may provide insights into the mechanisms responsible for membrane polarization, ion channel function, and activity of ionic pumps during the process of impulse conduction. IMI2-PainCare-BioPain-RCT1 hypothesizes that NET can serve as biomarkers of target engagement of analgesic drugs in this compartment of the nociceptive system for future Phase 1 clinical trials. Phase 2 and 3 clinical trials could also benefit from these tools for patient stratification.

**Trial registration:**

This trial was registered 25/06/2019 in EudraCT (2019-000942-36).

## Administrative information

Note: the numbers in curly brackets in this protocol refer to SPIRIT checklist item numbers. The order of the items has been modified to group similar items (see http://www.equator-network.org/reporting-guidelines/spirit-2013-statement-defining-standard-protocol-items-for-clinical-trials/).

**Table:** 

Title {1}	IMI2-PainCare-BioPain-RCT1: A randomized, subject and assessor blind, placebo-controlled, cross-over, multi-center trial in healthy subjects to investigate the effects of lacosamide, pregabalin and tapentadol on biomarkers of pain processing observed by peripheral nerve excitability testing (NET)
Trial registration {2a and 2b}.	EudraCT registration: 2019-000942-36
Protocol version {3}	3.0 (15/05/2019)
Funding {4}	This project has received funding from the Innovative Medicines Initiative 2 Joint undertaking under grant agreement No 777500. This Joint Undertaking receives support from the European Union’s Horizon 2020 research and innovation program and EFPIA.
Author details {5a}	Zahra Nochi1*, Hossein Pia1*, Petra Bloms-Funke2, Irmgard Boesl3, Ombretta Caspani4, Sonya C. Chapman5, Francesca Fardo1, Bernd Genser6, Marcus Goetz7, Anna V. Kostenko4, Caterina Leone8, Thomas Li9, André Mouraux10, Bernhard Pelz7, Esther Pogatzki-Zahn11, Andreas Schilder4, Erik Schnetter12, Karin Schubart13, Alexandre Stouffs10, Irene Tracey14, Iñaki F. Troconiz15, Andrea Truini8, Johannes Van Niel16, Jose Miguel Vela17, Katy Vincent18, Jan Vollert19, Vishvarani Wanigasekera14, Matthias Wittayer4, Hatice Tankisi20**, Nanna B. Finnerup1**c, Keith G Phillips21**, Rolf-Detlef Treede4** * shared first authorship, ** shared last authorship, c corresponding author 1.Danish Pain Research Center, Department of Clinical Medicine, Aarhus University, Aarhus, Denmark 2.Translational Science & Intelligence, Grünenthal GmbH, Aachen, Germany. 3.Clinical Science Development, Grünenthal GmbH, Aachen, Germany. 4.Department of Neurophysiology, Mannheim Center for Translational Neurosciences (MCTN), University of Heidelberg, Mannheim, Germany. 5.Eli Lilly and Company, Arlington Square, Bracknell, UK 6. Mannheim Institute of Public Health, Social & Preventive Medicine, University of Heidelberg, Germany 7.MRC Systems GmbH, Heidelberg, Germany. 8.Department of Human Neuroscience, Sapienza University, Rome, Italy. 9. Teva Branded Pharmaceutical Products R&D, Inc, West Chester, PA, USA 10. Institute of Neuroscience (IoNS), UCLouvain, Brussels, Belgium. 11.Department of Anaesthesiology, Intensive Care and Pain Medicine, University Hospital Münster, Münster, Germany. 12.University Computing Centre, University of Heidelberg, Heidelberg, Germany. 13.ConsulTech GmbH, Berlin, Germany 14.Wellcome Centre for Integrative Neuroimaging, FMRIB Centre, Nuffield Department of Clinical Neurosciences, University of Oxford, John Radcliffe Hospital, Oxford, OX3 9DU, United Kingdom 15.Department of Pharmaceutical Technology and Chemistry, School of Pharmacy and Nutrition, University of Navarra, Pamplona, Spain. 16.Mature Products Development, Grünenthal GmbH, Aachen, Germany. 17.Welab Barcelona, Barcelona, Spain. 18.Nuffield Department of Women's and Reproductive Health (NDWRH), University of Oxford, Oxford, UK. 19.Pain Research, Department of Surgery and Cancer, Imperial College London, London, UK. 20.Department of Clinical Medicine, Aarhus University and Department of Clinical Neurophysiology, Aarhus University Hospital, Aarhus Denmark 21.Neuroscience Next Generation Therapeutics, Eli Lilly and Company, Lilly Innovation Center, Cambridge, MA, USA
Name and contact information for the trial sponsor {5b}	Nanna Brix Finnerup, Danish Pain Research Center, Department of Clinical Medicine, Aarhus University, Palle Juul-Jensens Boulevard 165, DK-8200 Aarhus N, Denmark. Email: finnerup@clin.au.dk Tel: + 45 7846 3382
Role of sponsor {5c}	This study is one of four studies conducted in subtopic BioPain of the IMI2-PainCare project, coordinated by Rolf-Detlef Treede (Heidelberg University). Design of the study was led by the sponsor and coordinator, and involved all partners of the BioPain subtopic of the IMI2-PainCare consortium (WP5, WP6, WP7). The sponsor will coordinate data collection at all sites and extract all biomarker parameters from the source data. Final analysis of study endpoints will be coordinated by Rolf-Detlef Treede and involve all partners of the BioPain subtopic of the IMI2-PainCare consortium.

## Introduction

### Background and rationale {6a}

Chronic pain is the main cause of disability [1]. Treatment often provides inadequate pain relief or is associated with intolerable side effects. Few drugs have been developed for the treatment of chronic pain. Challenges in analgesic drug development include whether the drug under development engages the relevant pharmacological target to provide the expected effect and to what extent the drug reaches the target compartment (nerve, spinal cord or brain) at a sufficient concentration. The IMI-PainCare (http://imi-paincare.eu) is a research consortium with participants from 14 countries including academic institutions, European Federation of Pharmaceutical Industries and Associations (EFPIA), Small and medium-sized enterprises (SMEs), patient organizations and pain societies. The overall concept of the BioPain subtopic of IMI-PainCare has been described previously [2]. In short, we hypothesize that different biomarkers of the peripheral, spinal cord, and brain compartments of the nociceptive system can be used to assess drug exposure and target engagement to be used in the development of new analgesics and possibly also in clinical trials in pain patients.

### Objectives {7}

BioPain includes four similarly designed randomized controlled trials (RCT) using different biomarkers of peripheral nerve excitability (IMI2-PainCare-BioPain-RCT1)^1^, spinal cord and brainstem reflex activity (IMI2-PainCare-BioPain-RCT2)^2^, electroencephalographic measures of brain activity (IMI2-PainCare-BioPain-RCT3)^3^ [2], and functional magnetic resonance imaging measures of brain activity (IMI2-PainCare-BioPain-RCT4)^4^.

The objective of the overall BioPain has been presented previously [2]. IMI2-PainCare-BioPain-RCT1 will thus focus on biomarkers derived from peripheral nerve excitability testing (NET) [3–6]. The objective is to assess the effect of lacosamide, pregabalin, and tapentadol on large and small sensory and motor fibers using threshold tracking [3–7]. The assessment of small sensory afferents will be done using perception threshold tracking and will be done on both normal skin and in an area of sensitization using high-frequency electrical stimulation (HFS) on the skin, which causes sustained and reversible hyperalgesia due to sensitization [8, 9].

### Trial design {8}

**Fig. 1:**
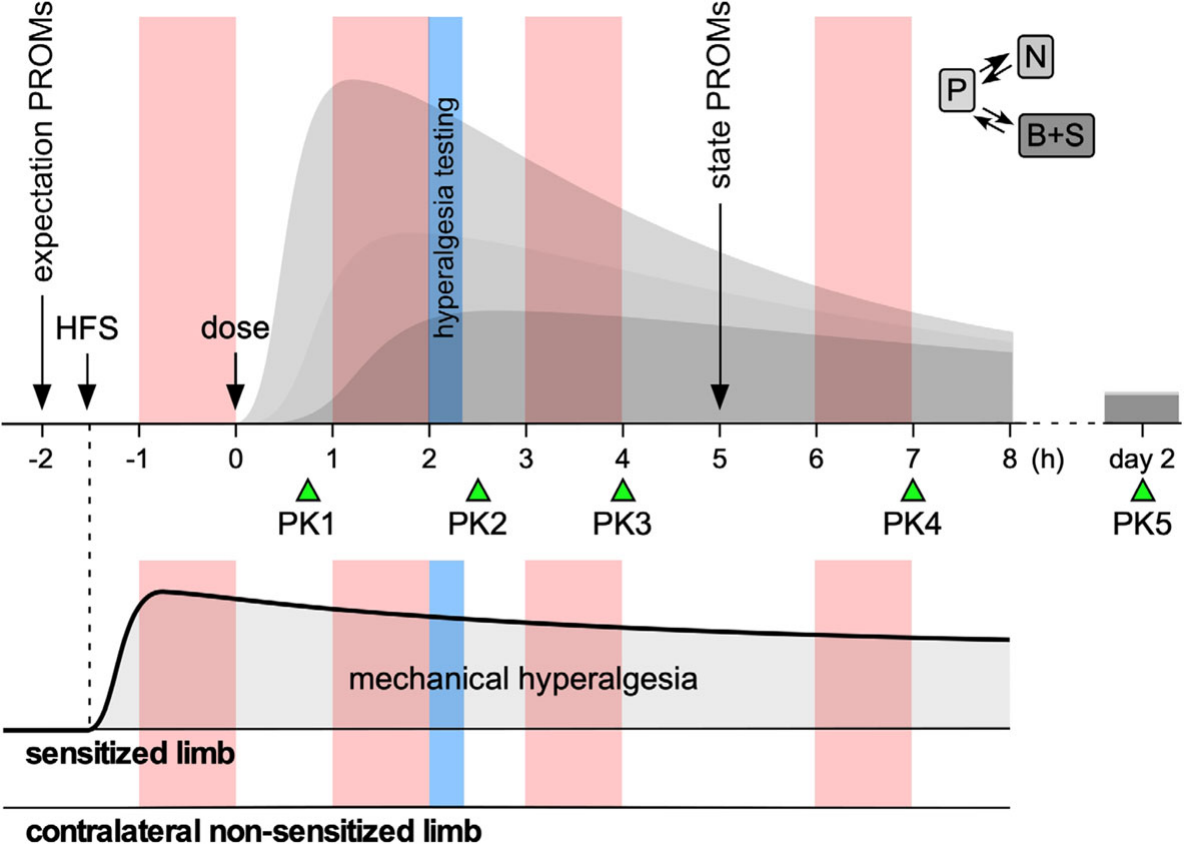
Trial design of each study period. After HFS, NET will be performed four times as indicated in light red. After the first test, the drug will be administered. Five blood samples will be taken to measure plasma drug levels (indicated by PK) and to model the PK profiles in the plasma (P), peripheral nerves (N), spinal (S), and brain (B) compartments. Patient-reported outcomes will be used to assess the subject’s expectations of pain, anxiety and pain relief (expectation PROMs), and tiredness and state anxiety (state PROMs). Assessment of hyperalgesia will be done once (light blue). Reproduced from [2]

IMI2-PainCare-BioPain-RCT1 is a multisite, single-dose, placebo-controlled subject- and assessor-blind, randomized 4-way crossover pharmacodynamics and pharmacokinetic study in healthy subjects. It is designed to test the hypothesis that the strength-duration time constant (SDTC) as an excitability parameter can serve as biomarker of target engagement of analgesic drugs in the peripheral compartment of the nociceptive system for future Phase 1 clinical trials. Two co-primary objectives and three key secondary objectives address the effects of lacosamide, pregabalin, and tapentadol on large motor fibers, large sensory fibers, and small sensory fibers (see below and Table 1). After completion of Patient-Reported Outcomes Measures (PROMS), HFS will be applied to the left forearm. Pharmacodynamic (PD) testing consisting of NET will be evaluated before and three times after a single dose of lacosamide, pregabalin, tapentadol, and placebo, given in four separate study periods separated by at least 1 week, as described previously for IMI2-PainCare-BioPain-RCT3 [2] (Fig. 1). NET of large sensory and motor fibers will be performed on the right non-sensitized wrist. Perception threshold tracking will be done bilateral on both the non-sensitized and sensitized forearm. Subjects are asked to rate the intensity of pain and unpleasantness after each PD (NET) sessions. PROMS will be collected additionally two times to assess subjective pain perception, and validated questionnaires will be used to assess psychological traits and states [2]. Five blood samples will be taken to measure plasma drug levels for pharmacokinetics.

**Table 1:** Primary and key secondary objectives

**Co-primary objectives:**	
1. To test if the SDTC changes (at planned first post-dose timing) of large sensory fibers differ in the lacosamide period as compared to the placebo period.	
2. To test if the SDTC changes (at planned first post-dose timing) of large motor fibers differs in the lacosamide period as compared to the placebo period.	
**Key secondary objectives:**	
1. To test if the SDTC changes (at planned first post-dose timing) of large sensory fibers differ in the pregabalin and/or tapentadol periods as compared to the placebo period.	
2. To test if the SDTC changes (at planned first post-dose timing) of large motor fibers in the pregabalin and/or tapentadol periods differs as compared to the placebo period.	
3. To test if the SDTC changes (at planned first post-dose timing) of small sensory fibers differ in the lacosamide period as compared to the placebo period.

## Methods: participants, interventions, and outcomes

### Study setting {9}

Four clinical sites in four countries will participate: The Danish Pain Research Center, Department of Clinical Medicine, Aarhus University (Sponsor and Principal Investigator: Nanna Finnerup), the Department of anesthesiology of the Cliniques universitaires Saint-Luc of the Université catholique de Louvain in Belgium (Principal Investigator: Patricia Lavand’homme), the Mannheim Center for Translational Neuroscience (MCTN) of the University of Heidelberg in Germany (Principal Investigator: Rolf-Detlef Treede), and the Department of Human Neuroscience of the Sapienza University of Rome in Italy (Principal Investigator: Andrea Truini). Details on the study sites can be obtained on the EudraCT clinical trials register (2019-000942-36).

All sites are academic hospitals and/or academic laboratories conducting research in human volunteers.

As presented previously for RCT3 [2], the following partners will have non-clinical roles in this study:

- Heidelberg University Computing Centre, Germany. Contribution: assuming responsibility for data storage and advanced statistical analysis.
- ConsulTech GmbH, Berlin, Germany. Contribution: ConsulTech will coordinate trial monitoring activities. Tasks include review and inspection of the quality of the data and the compliance to and implementation of regulations such as the declaration of Helsinki, ICH Good Clinical Practice (GCP) and the Clinical trial plan.
- MRC Systems (spin-off from the University of Heidelberg and the German Cancer Research Center in Heidelberg), Germany. Contribution: MRC Systems will provide to each clinical partner the multipin electrode used to deliver HFS, as well as the mechanical pinprick stimulators for the recording of hyperalgesia testing.
- Pharmacometrics and Systems Pharmacology (PSP), Department of Pharmacy and Pharmaceutical Technology of the School of Pharmacy, University of Navarra, Spain. Contribution: Integrate from a quantitative mechanistic and translational perspective, PK/PD information gathered from the study, as well as PK/PD information provided by preclinical in vitro and in vivo studies conducted within the BioPain subtopic of IMI-PainCare. The end-product will consist of a model formulated on the basis on the known and data-driven mechanisms of action that can be (among several other applications) (i) used through modelling and simulation to optimize dosing scenarios and (ii) applied retrospectively or prospectively in other scenarios to get meaningful PK/PD parameters.
- Grünenthal GmbH, Aachen, Germany. Contribution: Co-leading the task to support consensus on final study designs across IMI2-PainCare-BioPain-RCT1 to RCT4. Co-leading the task of clinical study implementation and operations.
- Eli Lilly and Company, research site Arlington Square, UK. Contribution: Co-leading the tasks of data delivery and analysis (preclinical and clinical), and preclinical biomarker back-translation, including PK. PK/PD/pharmacometric co-leadership and analysis support.
- WELAB Barcelona, Spain. Contribution: performing bio-analyses of the IMPs (Investigational medicinal products) as laid down in separate specification manuals.
- Teva Pharmaceutical Industries Ltd., headquartered in Petah Tikva, Israel. Contribution: pharmacometric support, clinical programming, data collection and capturing, and input of expertise related to CDISC.

### Eligibility criteria {10}

There will be an initial screening visit. Following this first screening of inclusion and exclusion criteria, the subject will be either excluded from the trial or scheduled for the first study period. Tables 2 and 3 list the inclusion and exclusion criteria at screening visit. These are identical across all four RCTs in BioPain and were previously published [2]

**Table 2:** Inclusion criteria at screening visit

	Inclusion criteria at screening visit	Justification / rationale
01	Provision of signed and dated informed consent form	Ethical requirement
02	Stated willingness to comply with all study procedures and regimens and availability for the duration of the study	Ethical requirement and to minimize dropout rate
03	Caucasian male or female subjects, aged 18 to 45 years	To minimize variability. Laser heat stimuli used to elicit LEPs will be delivered to the skin using an Nd:YAP laser. Because skin reflectance, absorption and transmittance of the infrared radiations generated by this laser are highly dependent on skin pigmentation, only Caucasian participants with light skin will be recruited.
04	Subjects must be in good health as determined by the medical history, physical and laboratory examinations and must not show any clinically significant deviations from reference ranges as determined by 12-lead electrocardiogram (ECG), vital signs (blood pressure, pulse rate and respiratory rate) and laboratory parameters (renal and hepatic function).	Subject safety and interpretability of results
05	Body mass index > 18 kg/m^2^ and < 30 kg/m^2^ with a minimum body weight of 45.0 kg and a maximum of 100 kg (for men and women)	Consistent with being in good health
06	Ability to take oral medication	Practical reason
07	For female subjects of childbearing potential: use of highly effective contraception with a low failure rate defined as < 1% per year for at least 1 month prior to screening and agreement to use such a method during study participation and for an additional 4 weeks after the end of study drug administration: - combined (estrogen and progestogen containing) hormonal contraception, - an intra-uterine device (hormone-free), - progestogen-only hormonal contraception associated with inhibition of ovulation, - an intra-uterine hormone releasing system (IUS) A woman of non-childbearing potential may be included if surgically sterile (i.e., after laparoscopic or hysteroscopic sterilization, hysterectomy or bilateral oophorectomy) or post- menopausal for at least 2 years.	To avoid pregnancies with potential harm to the unborn
08	Right hand dominance (assessed using the Edinburgh Handedness Inventory, and defined as a score ≥ 60)	To minimize variability

**Table 3:** Exclusion criteria at screening visit

	Exclusion criteria at screening visit	Justification / rationale
01	Presence of any medical devices (e.g., cardiac pacemaker), implants or prothesis unless it is beyond discussion that these will not put the subject’s safety during the study at risk and will not interfere with the results of the study.	To avoid interference with the purpose of the study and to ascertain the subject’s good health
02	Known or suspected allergic reactions or hypersensitivity to components of lacosamide (Vimpat®). Second or third degree atrioventricular (AV) block.	Contraindications for lacosamide
03	Known or suspected allergic reactions or hypersensitivity to components of pregabalin (Lyrica®).	Contraindications for pregabalin
04	Known or suspected allergic reactions or hypersensitivity to components of tapentadol (Palexia®). Known contraindication for drugs with μ-opioid agonist activity, i.e., significant respiratory depression, acute or severe bronchial asthma or hypercapnia. Present or suspected paralytic ileus. Acute intoxication with alcohol, hypnotics, centrally acting analgesics, or psychotropic drugs.	Contraindications for tapentadol
05	Not willing or able to abstain from changes in physical exercise activities during the Study.	To avoid interference with the purpose of the study
06	Any chronic pain condition or recent (i.e., within the preceding 2 years) history thereof.	To avoid interference with the purpose of the study
07	Migraine (at least 1 attack in the last 24 months)	To avoid interference with the purpose of the study
08	Recurrent headache or back pain on more than 5 days/month in the last 3 months	To avoid interference with the purpose of the study
09	Caffeine consumption of more than 8 servings of coffee, tea, or other caffeinated drinks per day. Each serving is approximately 120 mg of caffeine	To avoid interference with the purpose of the study
10	Any relevant symptom of neurological dysfunction of the motor and sensory system that may interfere with the conduct of the study.	To avoid interference with the purpose of the study
11	Clinically evident psychiatric diseases (e.g., depression, anxiety).	To avoid interference with the purpose of the study
12	History or symptoms of central nervous system disease or peripheral nerve lesions or dysfunction with sequelae that may impact the study assessments or that may deteriorate by one dose of a drug with antiepileptic, noradrenergic or opioid activity.	To avoid interference with the purpose of the study Subject safety
13	Focused neurological examination showing signs of abnormality.	To avoid interference with the purpose of the study
14	Active internal disease or sequelae of internal disease (e.g., diabetes mellitus, liver diseases, kidney diseases, cardiovascular diseases, hypo- or hyperthyroidism, hypertension).	To ascertain the subject’s good health
15	Diseases or conditions known to interfere with the distribution, metabolism, or excretion of drugs.	To avoid artifacts
16	Clinically significant disease (e.g., medical history of infection with human immunodeficiency virus (HIV) type 1 or type 2, hepatitis B, or hepatitis C) or condition that may affect efficacy or safety assessments, or any other reasons which, in investigator’s opinion, may preclude the subject’s participation in the trial.	Safety of investigator and their staff Standardization of the trial population
17	Not willing or able to abstain from alcohol from 48 h prior to any study period and until the end of the study period.	To ascertain and protect the subject’s good health and suitability for the study
18	Consumption of cannabis in the last 4 weeks prior to the study.	To ascertain and protect the subject’s good health and suitability for the study
19	Evidence or history of alcohol or drug (opioids, amphetamines, benzodiazepines cannabinoids) abuse (as defined by ICD-10 or DSM IV) including positive or missing drugs of abuse screen (urine drugs of abuse test). Consumption of more than 21 alcohol units per week for male subjects and more than 14 units per week for female subjects (1 alcohol unit = 1 beer [12 oz/355 mL] = 1 wine [5 oz/150 mL] = 1 liquor [1.5 oz/40 mL] = 0.75 oz/20 mL alcohol).	To ascertain and protect the subject’s good health and suitability for the study
20	Habitually smoking more than 10 cigarettes, 2 cigars, or 2 pipes of tobacco per day within the last 6 months before enrollment in this trial.	To ascertain the subject’s good health
21	Known or suspected of not being willing or able to comply with the requirements of the trial protocol or the instructions.	To ascertain the subject’s suitability for the study
22	Inability to communicate meaningfully with the trial site staff (e.g., insufficient language skills).	To ascertain the subject´s safety
23	Any person with direct involvement in the trial conduct; any person under the direct supervision of the investigator or dependent on the investigator.	Ethical requirement
24	Blood loss of 500 mL or more (e.g., owing to blood donation) within 3 months before enrollment in this trial.	To ascertain the subject’s suitability for the study
25	Pregnancy, planned pregnancy or lactation.	Ethical requirement to protect the unborn or newborn child
26	Presence of dermatological conditions in the test areas of the study that would prevent the proper application of study procedures, such as electrodes for HFS, pinprick (dermatitis, psoriasis, contact eczema, local changes of the skin due to regularly playing volleyball, etc.).	To avoid interference with the purpose of the study
27	Any other reason to exclude the subject according to judgment by the investigator	To avoid interference with the purpose of the study.
A set of *temporary exclusion criteria* have also been defined. The subject will not be excluded if some of these temporary exclusion criteria are met during the screening visit. Instead, the first study period may be postponed. Before the start of the first study period, previously met temporary exclusion criteria will be checked again, and their absence will be verified before the screening for the first study period takes place.		
28	Any drug intake in the past 2 weeks including antibiotics, herbal medicines and other remedies except the following allowed drugs: oral paracetamol or ibuprofen for a self-limiting condition (e.g., toothache, bruise) for up to 3 days in total within the past 2 weeks; oral antihistaminics and nasal aerosol and topical treatments for seasonal allergy up to 1 week before screening; contraceptives are allowed without time limit.	To ascertain the subject’s good health and to avoid interference with the purpose of the study
29	Any transient illness within 2 weeks before screening.	To ensure the subject’s good health
30	Changes in physical exercise activities, e.g., starting workout/training within 1 week before screening.	To avoid interference with the purpose of the study
31	Current or recent (during the preceding 2 weeks) acute pain lasting more than 4 h.	To avoid interference with the purpose of the study
32	Jet lag / irregular working hours / sleep restriction in the last 3 days before the screening period.	To avoid interference with the purpose of the study

Reproduced from [2]

### Who will take informed consent? {26a}

This is identical across all four RCTs and was previously described [2]. In short, informed consent will be obtained by the Principal Investigator or authorized trial site staff before any trial-related procedures following the GCP guidelines and applicable regulatory requirements. The information sheet and informed consent form will be approved by the relevant Independent Ethical Committees (IECs).

### Additional consent provisions for collection and use of participant data and biological specimens {26b}

The informed consent will explain how participant data and blood samples will be handled, where they will be sent and that BioPain partners will have access to pseudoanonymized data and explicitly obtaining the subjects’ consent for this sharing. In addition, the possibility that anonymized samples and data could be shared by the Investigator with third parties will be mentioned and explicitly obtaining the subjects’ consent for this sharing.

## Interventions

### Explanation for the choice of comparators {6b}

The rationale for the chosen investigational medicinal products (IMPs) and their dose have been described previously for RCT3 [2] and will be briefly summarized here.

#### Lacosamide

Lacosamide has marketing authorization for epilepsy in the EU. Animal studies have suggested an effect on pain-like behavior in neuropathic models [10] and although clinical RCTs have inconsistent results [11–15], effects were shown for lacosamide 400 mg daily in painful diabetic neuropathy [13, 14] and small fiber neuropathy due to na_v_1.7 mutations [15]. Lacosamide has been shown to normalize the firing pattern of C fibers using microneurography and revert abnormal excitability of nociceptors derived from human-induced pluripotent stem cells, suggesting a specific modification of the function of peripheral nociceptors [16]. Single oral doses of 200 mg lacosamide have reportedly been administered to healthy subjects [17, 18] with acceptable side effects such as dizziness, tiredness, fatigue, paresthesia surrounding the mouth, and thrombophlebitis.

#### Pregabalin

In the EU, pregabalin has marketing authorization for the treatment of peripheral and central neuropathic pain in adults in doses of 150 to 600 mg per day. Single oral doses of 300 mg pregabalin have been administered to almost 200 healthy subjects [19–22]. Side effects included dizziness, somnolence, fatigue, and euphoric mood and no subject was withdrawn from the study for safety reasons.

#### Tapentadol

Tapentadol sustained release is indicated in the EU for the management of severe chronic pain in adults, which can be adequately managed only with opioid analgesics. Tapentadol immediate release (film-coated tablets) is indicated in the EU for the relief of moderate to severe acute pain in adults, which can be adequately managed only with opioid analgesics. Two randomized withdrawal trials indicated the effectiveness of tapentadol for painful diabetic polyneuropathy [23, 24]. The 100 mg tapentadol administered to healthy subjects is referred to as highest therapeutic dose. Side effects include nausea, dizziness, drowsiness, and headache.

#### Placebo

Placebo serves to minimize bias as to act as a control condition.

Given the three drugs and their side effects, the following harms during a single drug treatment are expected: temporary and reversible dizziness, tiredness, somnolence, euphoric mood, nausea, and headache.

### Rationale for the induction of hyperalgesia using high-frequency stimulation (HFS)

High-frequency electrical pulses delivered to the skin using a multipin electrode designed to preferentially activate cutaneous nociceptors is a validated and non-invasive procedure to induce a stable secondary hyperalgesia surrounding the location where HFS was applied due to central sensitization lasting at least 4 h [8, 9, 25]. We will assess the area of secondary hyperalgesia to pinprick and the intensity of pinprick hyperalgesia and dynamical mechanical allodynia. HFS induces a local skin flare response but does not cause long-lasting spontaneous pain.

### Intervention description {11a}

The IMPs will be (1) lacosamide (Vimpat®) film-coated tablets (composition: 2 × 100 mg lacosamide tablets); (2) pregabalin (Lyrica®) capsules (composition: 2 × 75 mg pregabalin capsules); (3) tapentadol (Palexia®) immediate release tablets (composition: 2 × 50 mg tapentadol immediate release tablet); and placebo capsules (composition: 2 × hard gelatine capsules filled with mannitol and colloidal silicon dioxide) (2).

Each IMP will be overencapsulated and administered as single oral dose (two capsules), in a double-blind, 4-period, crossover fashion where the study periods are separated by at least 1 week. All IMPs, except placebo, are registered medications in the countries that will participate in the trial. IMPs will be obtained from commercial stock [2].

As described for RCT3 [2], for the induction of hyperalgesia, HFS will be delivered to superficial nerve terminals using a multipin surface electrode similar to the electrode used in Klein et al. [8] and developed by MRC. The stimuli will be applied to the skin of the left volar forearm. The electrical pulses will be generated by a standard, CE-approved, constant-current electrical stimulator routinely used for clinical diagnostic purposes. The stimulation will consist in trains of 100-Hz pulses lasting 1 s and repeated five times, at an intensity sufficient to generate strong activity in small-diameter nociceptive afferents.

### Criteria for discontinuing or modifying allocated interventions {11b}

At the start of each of the four study periods, subjects will be excluded from the period if any of the criteria listed in Table 4 apply [2].

**Table 4:** Exclusion criteria at study periods

	Exclusion criteria at study periods:	Justification / rationale
33	For female subjects of child bearing potential: positive or missing pregnancy test	To protect a fetus
34	Positive or missing urine test for drugs of abuse (opioids, amphetamines, benzodiazepines, cannabinoids).	Subject safety and to avoid interactions with, e.g., tapentadol (PD interactions, safety interactions)
35	Blood loss of 500 mL or more (e.g., owing to blood donation) since screening.	To ascertain the subject’s suitability for the study
36	Any other reason to exclude the subject according to judgment by the investigator	To avoid interference with the purpose of the study.
Temporary exclusion criteria at study periods. The subject is not excluded if some of these temporary exclusion criteria are met at screening of the study period. Instead, the study period may be postponed. If this is the case, all temporary exclusion criteria will be checked again.		
37	Alcohol consumption in the last 48 hours prior to the study period.	Subject safety and to avoid interactions with, e.g., tapentadol (PD interactions, safety interactions)
38	Intake of any drug including herbal medicines and other remedies except the following: contraceptives; oral paracetamol or ibuprofen up to the maximum recommended dose according SmPC, with last intake for both > 4 days prior to each study period for a self-resolving condition.	As described for screening visit
39	Changes in physical exercise activities, e.g., starting workout/training within 1 week prior to the study.	To avoid interference with the purpose of the study
40	Current pain within the last 4 days before the study period.	To avoid interference with the purpose of the study
41	Any transient, clinically relevant illness within 4 days before the period.	To ensure the subject’s good health
42	Incidentally not willing or able to comply with the requirements of the trial protocol or the instructions or to communicate meaningfully with the trial site staff.	To ascertain the subject’s suitability for the study
43	Incidentally unable to take oral medication.	Requirement for the study
44	Jet lag / irregular working hours / sleep restriction in the last 3 days before the period.	To avoid interference with the purpose of the study

Reproduced from [2]

### Strategies to improve adherence to interventions {11c}

At each study period, an oral dose of lacosamide, pregabalin, tapentadol, or placebo will be taken with 100 mL of plain water [2]. After intake of the study medication, the investigator will inspect the subject’s mouth to verify that the medication is swallowed.

### Relevant concomitant care permitted or prohibited during the trial {11d}

Prohibited drugs at screening and for each study period is described in Table 3 (Exclusion Criterion #28) and 3 (Exclusion Criterion #38), respectively.

### Provisions for post-trial care {30}

Suitable insurance for subjects will be in place at each site. A follow-up telephone call will be made between 7 and 14 days after the last study period to ensure the absence of adverse events. Otherwise, no post-trial care is foreseen as the study is conducted in healthy volunteers.

### Outcomes {12}

The objective of the study is to evaluate the effect of the study drugs on a set of biomarkers derived from peripheral nerve excitability.

### Rationale for the chosen biomarkers

Measurements of peripheral nerve excitability using threshold-tracking protocols are sensitive to membrane potential at the site of stimulation. Set of useful indices of axonal excitability collectively may provide insights into the mechanisms responsible for membrane polarization, ion channel function, and activity of ionic pumps during the process of impulse conduction [3, 4]. Standard NET assesses the function of large fibers. The nerve is stimulated directly, and the compound action potential is measured distally. Perception threshold tracking is a method where the membrane potential is assessed distally in the nerve fiber. With the use of small cathodes, the small fibers can be assessed. The subject will press a button when he/she feels a sensation, and the perception threshold is tracked.

### Primary and key secondary outcomes

The primary endpoints are the changes of the strength-duration time constant (SDTC) measured in large sensory fibers and in large motor fibers, both at the planned first PD time point post dosing relative to their pre-dose PD measurement (i.e., difference to period specific baseline). The SDTC is a measure for the dependency of the threshold current intensity related to the duration of a stimulus. These endpoints are determined four times per subject, once per period (i.e., once per different IMP administered). The primary analysis will concentrate on the comparison of the lacosamide vs. placebo effect, while the primary endpoints will also be tested in key secondary analyses. Lacosamide was chosen for primary outcome, as we expect this drug to act on the peripheral compartment that is the compartment assessed in RCT1.

The key secondary endpoint is the SDTC measured in small sensory fibers at the planned first PD time point post dosing relative to the pre-dose PD measurement (i.e., difference to period specific baseline).

### Definition of other prespecified analyses

- Percentage change in motor and sensory threshold electrotonus (the amount of change in membrane excitability in response to long-lasting preceding de- or hyperpolarizing currents) (TEh (90–100 ms), TEh (peak, − 70%) and S2 accomodation) vs. baseline
- Change in refractoriness and relative refractory period in recovery cycle of nerve excitability (motor and sensory large fibers)
- *PK/PD analysis*. As both drug concentrations and biomarker responses are measured at several time points post drug administration, the relationship between drug levels and select biomarkers will be explored, and modeled

There are many other exploratory analyses and outcomes corresponding to the variety of drugs and time points when assessments are done, the collection of pain intensity and PROMs, etc. These are described in more detail in the statistical analysis plan.

### Participant timeline {13}

**Fig. 2:**
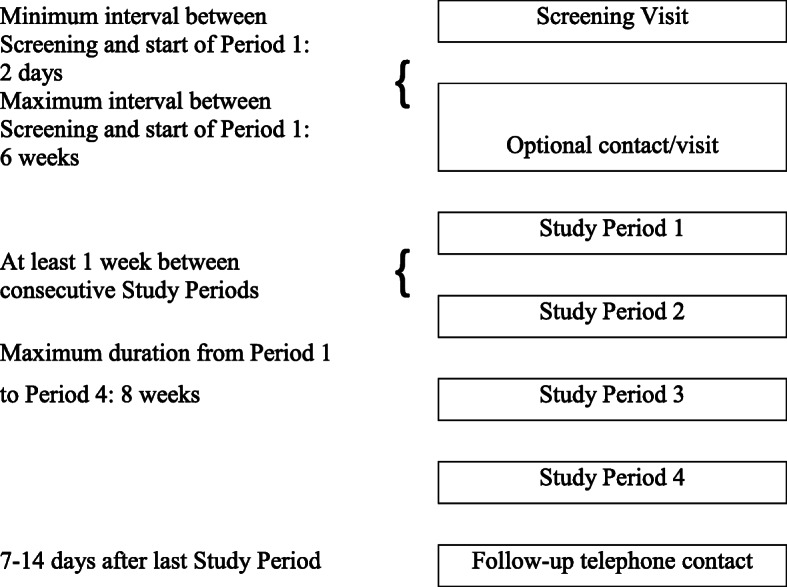
Timeline of the study which includes a screening visit, an optional contact, four study periods separated by at least 1 week, and a follow-up telephone contact. Reproduced from [2]

Figure 2 summarizes the participant timeline, which includes a screening visit, followed by four study periods and a follow-up telephone contact similar to RCT3 [2]. There will be an optional contact before the first study period. If screening of exclusion criteria for eligibility for period 1 shows that one or more temporary exclusion criteria are met, the start of period 1 can be postponed and re-scheduled [2]. Each subject is expected to be in the trial for approximately a minimum of 30 days and a maximum of 14 weeks.

### Screening visit

As described previously for RCT3 [2], the following will be performed:

- Explain the purpose of the research, the extent and burden of the procedures and assessments.
- Obtain informed consent.
- Assess subject handedness using the Edinburgh Handedness Inventory.
- Record demographic data.
- Record prior and concomitant medication.
- Record clinically relevant medical and surgical history.
- Assess inclusion criteria.
- Perform a focused neurological examination in the presence of any clinically evident sensory disorder and a physical examination if indicated from the medical history.
- Record a 12-lead electrocardiogram and verify absence of signs of second or third degree atrioventricular block.
- Perform urine pregnancy test.
- Collect a blood sample to verify normal renal and hepatic functions.
- Perform urine test for drug abuse (opioids, amphetamines, cannabinoids) and perform alcohol consumption check.
- Record psychosocial characteristics using patient-reported outcome measures and validated questionnaires.
- Instruct the subject on the study-specific procedures including how to use the rating scales.
- Demonstrate the test stimuli that will be used, induce sensitization at the left forearm using HFS and perform hyperalgesia testing 20 min after induction.
- Assess exclusion criteria specific for the screening visit.

### Optional contact before start of treatment period

The optional (telephone / email) contact will be after the screening visit and at the latest 48 h before the first study period with the purpose to arrange the time for the first study period and remind the subject of restrictions and ensure the subject had not taken prohibited drugs.

### Treatment periods: study periods 1, 2, 3, and 4

Each study period will be separated by at least 1 week. The subjects will have a light breakfast at home. The schedule of events is identical for all study periods and is provided in Table 5 and presented previously for RCT3 [2].

**Table 5:** Detailed timetable of procedures and assessments in periods 1, 2, 3, and 4

Clock time	Time relative to dose (minutes)	Time relative to HFS (minutes)	Dose	HFS	PK	PD	Hyperalgesia testing**	PROMs	(D)rink (M)eal
08:00	− 150	− 60							
08:30	− 120	− 30						X	
09:00	− 90	0		X					
09:30	− 60	30				[1]			
10:00	− 30	60							
10:30	0	90	X						D
11:00	30	120							
11:15	45	135			[1]				
11:30	60	150				[2]			
12:00	90	180							
12:30	120	210					X		D
13:00	150	240			[2]				
13:30	180	270				[3]			
14:00	210	300							
14:30	240	330			[3]				M
15:00	270	360							
15:30	300	390						X	
16:00	330	420							
16:30	360	450				[4]			
17:00	390	480							
17:30	420	510			[4]				D
18:00	450	540							
Next day					(5)*				

*The PK sample on next day can be taken at any suitable time provided that the exact time of sampling is precisely recorded. **Hyperalgesia testing at the sensitized and contralateral forearm, harmonized across all four IMI2-PainCare-BioPain RCTs. Reproduced from [2]

Procedures, assessments, and events during a period as described previously for RCT3 [2]:

- The subject will have breakfast at home and arrive at the site at or before 08:00 AM.
- Record prior and concomitant medication.
- Urine screening test for drugs of abuse and alcohol consumption check.
- For female subjects: urine pregnancy test.
- Reassess subject eligibility for the study according to inclusion and exclusion criteria.
- Train / instruct again the subject on the study-specific procedures.
- Complete PROMs assessing subject expectations.
- Optionally, according to local practices, an indwelling venous catheter will be inserted at the start of each study period and will be left in place for the duration of the study day.
- HFS will be applied to the left forearm to induce sensitization.
- IMP administration.
- A total of 5 blood samples (6 mL each) will be taken as scheduled in Table 5 for pharmacokinetic (PK) analyses. The last sample will be taken on the next day, at any suitable time.
- One pre-dose and 3 post-dose PD biomarker assessments will be made as scheduled in Table 5.
- Hyperalgesia testing at the sensitized and contralateral forearm will be made as scheduled in Table 5, harmonized across all four IMI2-PainCare-BioPain-RCTs.
- Complete PROMs assessing tiredness and anxiety.
- Drinks (water or sugared juice, e.g. apple juice) and a light meal will be served as scheduled in Table 5.
- Instruct the subject not to drive or bike or operate machinery for the entire day (risk of sedation or dizziness caused by IMP). Instruct the participants that they should not drive or bike or operate machinery on the following day if they feel drowsy or dizzy.
- Upon leaving the trial site, if the subject is feeling drowsy or dizzy, arrange for the participant to be driven home by taxi.

### Follow-up telephone call

Between 7 and 14 days after the end of the last study period, the absence of untoward medical or mental sequelae of the study will be ascertained in a follow-up telephone call with the subject [2].

### Sample size {14}

**Fig. 3:**
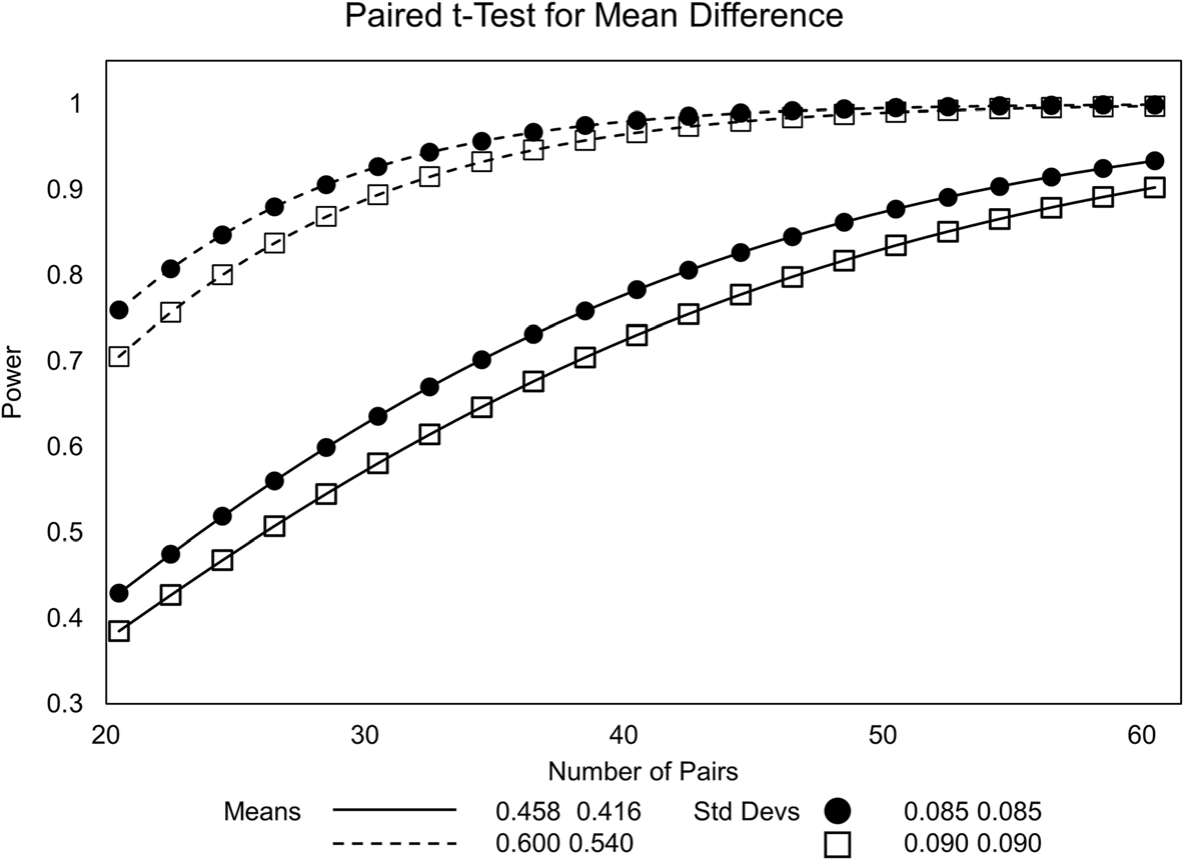
Power as a function of number of pairs (paired *t*-test for mean difference)

Knowledge on the variability and effect sizes of excitability parameters is available in amyotrophic lateral sclerosis (ALS) patients with retigabine as treatment [26] as well as in neuropathic pain patients and healthy controls with mexiletine as treatment [27]. Both studies identified group differences between 0.04 and 0.06 ms (0.416 vs. 0.458 on motor nerve excitability [26] and 0.54 vs. 0.60 on sensory nerve measures [27], respectively) and standard deviations around 0.085 (but potentially as high as 0.10) [26, 27]. With the lacosamide vs. placebo comparison of the primary analysis (2-sided type I error of *α*/2 = 0.025) being essentially a paired *t*-test (assumed correlation of 0.5), the following power graphic yields a reasonable sample size, i.e., power > 80% (STATA, StataCorp LCC, USA). The study on large sensory fibers yielded much higher power than the study on motor fibers (Fig. 3, dashed vs. drawn lines), while the range of expected standard deviations had only small effects on power (crosses vs. circles). Based on the worst case, a power of 0.80 would require 48 subjects, while in the best case scenario 22 subjects would be sufficient.

To compensate for early dropouts, e.g., during the first period, we plan to randomize a total of 60 subjects (in the sense of being randomized for entering the treatment phase).

### Recruitment {15}

Study participants will be recruited by advertisements on webpages, newspapers, university billboards, etc. Subjects will be pre-screened to identify who could potentially be enrolled in the trial.

#### Screen failures

As presented for RCT3 previously [2], screen failures are defined as subjects who consent to participate in the clinical trial but are not subsequently randomly assigned to the IMP. A minimal set of screen failure information is required to ensure transparent reporting of screen failure subjects, to meet the Consolidated Standards of Reporting Trials (CONSORT) publishing requirements and to respond to queries from regulatory authorities. Minimal information includes demography, screen failure details, eligibility criteria, and any serious adverse event (SAE).

#### Subject discontinuation from the trial

As presented for RCT3 previously [2], once a subject enrolls in the trial, the trial site will make every effort to retain the subject for the planned duration of the trial, while protecting subject safety. A subject may withdraw consent at any time. This will automatically lead to discontinuation of the subject from the trial. The investigator will discontinue the subject’s participation in the study if further participation would involve unjustifiable risk to the subject’s mental or physical well-being or if participation would be against the purpose and interest of the study. In general, subjects who discontinue are those who complete the end of trial earlier than the end of study period 4. Subjects who discontinue their participation in the trial will not be replaced. The Principal Investigator will document on the case report form (CRF) any discontinuation of a subject and inform the sponsor. Where applicable, the relevant IEC(s) must be informed with a detailed written explanation.

The following will be done for all discontinued subjects, including those who withdrew informed consent as described previously [2]:

- Document the main reason for discontinuation from the trial.
- Ensure that all data collected until the time of discontinuation is transferred to the CRF.
- Complete any other trial-related formalities, e.g., those related to discharge from the trial site.
- For subjects withdrawing consent, document in the source data, the date, and time of withdrawal.
- If a subject withdraws consent and agrees (documented in writing), conduct the follow-up telephone call.

## Assignment of interventions: allocation

### Sequence generation {16a}

Randomization will be done blind to investigators as described previously [2] and will consist of blocks of four “4-period sequences,” these sequences being random permutations of the four 4-period sequences of a (basic) Latin square [28]. Randomization will be by site. At the first study period day before first IMP administration, subjects will be randomized to receive the lowest available randomization number at the site.

### Concealment mechanism {16b}

Each IMP will be overencapsulated and will be administered as single oral dose (two capsules). A sealed decoding envelope per treatment period will be provided for each randomization number. Each envelope will contain the identification of the IMP allocated to that subject [2].

### Implementation {16c}

The IMPs are purchased by the Heidelberg University Hospital Pharmacy that will overencapsulate the IMPs, manufacture the placebo, and generate allocation sequence and sealed envelopes in compliance with applicable local regulations. Detailed information is reported in a specification document (available on request). Labels will specify storage conditions and the IMPs will be stored in a secure place with restricted access and temperature monitoring. All sites will be licensed according to local laws for the receipt, storage, handling, and administration of narcotics as described previously [2]. Unblinding will be done by the statistician after study completion and the database is locked. The sealed code envelope is only unsealed/opened in emergency cases if safety of the subject requires knowledge of the treatment given.

## Assignment of interventions: Blinding

### Who will be blinded {17a}

As described for RCT3 [2], the investigator/trial personnel and subjects will be blinded to the assignment of pregabalin, tapentadol, lacosamide, and placebo (double-blind procedure). The personnel analyzing the plasma samples for PK analysis will be unblinded during the bioanalytical analysis, but will supply their data to the trial database in a blinded fashion.

### Procedure for unblinding if needed {17b}

If a sealed decoding envelope needs to be opened in an emergency case, the reason and time of unblinding as well as the person performing the unblinding will be documented [2].

## Data collection and management

### Plans for assessment and collection of outcomes {18a}

#### Collection of pharmacokinetic data

In each study period, 4 blood samples, 6 mL each, will be drawn on the day of drug dosing and 1 blood sample will be drawn the following day into tubes containing K2-EDTA as anticoagulant and will be centrifuged within 30 min after collection [2]. Thus, in total, 132 ml will be drawn per subject over the four study periods. As described for RCT3 [2], the harvested plasma will be frozen at − 20 to − 80 °C within 1 h of sampling and kept frozen until sent for analysis. Details will be provided in a separate specification manual. Bioanalysis will be performed by Welab using validated methods.

#### Collection of demographic data and other baseline characteristics

Demographic data, concomitant medication, and medical history will be collected as described for RCT3 [2] and in the inclusion and exclusion criteria. In case of a clinical relevant sensory disorder, a focused neurological examination will be performed at screening. At screening, a 12-lead electrocardiogram (ECG) will be recorded, a urine pregnancy test and drug abuse test will be performed, abstinence of consumption of alcohol will be checked, and a blood sample will be collected to verify normal renal and hepatic functions [2].

#### Collection of pharmacodynamic data

In each PD session, the skin is cleaned and electrodes are placed. First, perception threshold tracking is performed on the right (non-sensitized) forearm. Then motor and then sensory NET are performed on the right wrist. Finally, perception threshold tracking is performed on the left (sensitized) forearm.

*Motor and sensory nerve excitability tests*

Motor and sensory nerve NET will be performed on the median nerve at the wrist using a computerized program QtracW (Institute of Neurology, University College London, distributed by Digitimer Ltd) as described previously [3]. For this study, modified shorter protocols, TRONDOLM and TRONDOLS for motor and sensory NET, respectively, will be used. It will be performed on the right wrist, i.e., the wrist contralateral to where HFS is applied. Multiple excitability parameters will be assessed including [1] stimulus response curve to define the amplitude of the target response, [2] SDTC, a measure of passive membrane properties and nodal persistent Na+ conductance [3]; threshold electrotonus, a measure of internodal conductances and membrane potential, and [4] recovery cycle of excitability, an assessment of the recovery of excitability following an action potential marking the function of nodal Na+ channels.

*Small fiber nerve excitability tests*

To assess small fiber nerve excitability, perception threshold tracking will be performed using the TRONDRT4B protocol. This will be examined bilaterally, i.e., on both sensitized and non-sensitized forearms. An electrode with small cathodes (the same electrode that is used for HFS) will be placed on the skin. The perception threshold is estimated by increasing the intensity of repeated stimulations until the subject feels the stimulation, which is indicated by a button press. Since the subject indicates when a stimulation is barely perceptible, the sensations are typically non-painful. Stimulus response curves and SDTC are the excitability parameters that can be obtained by perception threshold tracking.

The total duration of the assessment will be 60 min. During the entire assessment, subjects will be seated in a comfortable chair. Details are given in an operational manual.

### Non-NET-derived pharmacodynamic data

#### Pain intensity and unpleasantness ratings

Participants will rate the intensity of pain and unpleasantness elicited by each PD session on a 0–100 numeric rating scale.

#### Patient-reported outcome measures (PROMs)

PROMs will be collected via questionnaires in the local language. The following PROMs will be completed during the screening visit, in the following order as described for RCT3 [2]: (1) the PROMIS Global-10 questionnaire, which consists of 10 items that assess general domains of health and functioning including overall physical health, mental health, social health, pain, fatigue, and overall perceived quality of life, (2) self-efficacy using 3 items of the General Self-Efficacy Scale (GSE) that assesses optimistic self-beliefs [29], (3) the General Anxiety Disorder-7 questionnaire (GAD-7) assessing trait anxiety, (4) the Patient Health Questionnaire-9 (PHQ-9) that assesses depressive symptoms, (5) the Pain Catastrophizing Scale (PCS) [30], (5) the Pain Sensitivity Questionnaire (PSQ) [31].

The following PROMs assessing subject expectations will be completed at the beginning of each study period as described for RCT3 [2]: (1) Anxiety will be assessed by asking participants: “On a scale of 0–100, please rate, how anxious you are about the upcoming experiment, with 0 being ‘not anxious at all’ and 100 being ‘extremely anxious’”, (2) Pain expectation will be assessed by asking participants: “On a scale of 0-100, please rate how much pain do you anticipate experiencing during the upcoming experiment, with 0 being ‘no pain at all’ and 100 being ‘pain as bad as you can imagine’,” and (3) Expectation of IMP-induced pain relief will be assessed by asking participants: “On a scale from 0 to 100, please rate how much pain relief you expect from the medication, with 0 being ‘expecting no relief’ and 100 being ‘expecting complete relief’.”

PROMs assessing tiredness on a 0–100 scale and state anxiety using the State-Trait Anxiety Inventory (STAI, by Mind Garden, Inc©) [32] will be assessed 5 h after IMP administration.

#### Hyperalgesia testing

The intensity of the sensation elicited by calibrated mechanical pinprick and brush stimuli will be assessed at the left (HFS-sensitized) and right (non-sensitized) forearms using a 0–100 numeric rating scale. At the sensitized forearm, the extent of the area of secondary hyperalgesia and allodynia will be measured along 8 radial directions from where the HFS was previously applied.

### Plans to promote participant retention and complete follow-up {18b}

To promote participant retention, we allowed that subjects can be included at a later timepoint if temporary exclusion criteria, such as transient illness or intake of medication were met. In addition, we allowed up to 8 weeks from study period 1 to 4 allowing for some periods to be separated by more than 1 week.

### Data management {19}

Data management will be performed by the Heidelberg University Computing Centre. The data management process is detailed in a data management plan. As described for RCT3 [2], all source data will be kept by the investigator, who will provide direct access for trial-related monitoring, audits, ethics committee review, and regulatory inspections. Paper source sheets for each subject will be provided to the investigator in electronic format and will serve to create the local source documentation in paper format and subsequently data will be entered into an electronic CRF system via a secured access to the Research Electronic Data Capture (REDCap) database hosted at the Heidelberg University computing center using double-entry by investigator or local authorized personnel. Entries will be checked against appropriate source documents by authorized CRAs as deemed appropriate in the monitoring guidelines as described for RCT3 [2]. Pharmacodynamic data from all sites will also be transferred to the Computing Center of the University of Heidelberg and derived endpoint readouts will be extracted centrally at Aarhus University where the data will be stored on secured servers. Derived data will be transferred to the relational database hosted at the Heidelberg University computing center. Pharmacokinetic concentrations and pharmacokinetic parameters measured by Welab will be uploaded by Welab to the relational database at the Heidelberg University computing center. Investigator site file and trial master files will be kept according to GCP.

### Confidentiality {27}

Data that can identify the subjects will be kept in accordance with applicable regulatory requirements at the local site. The data transferred will be pseudoanonymized with a code but will otherwise not contain any data that can identify the individual persons such as name.

### Plans for collection, laboratory evaluation, and storage of biological specimens for genetic or molecular analysis in this trial/future use {33}

This is described in detail for RCT3 [2] and is summarized here. The analysis of drug levels will be done from all samples while placebo samples will be analyzed at a single time point around *t*_max_ of drugs by Welab ADME Development Department using a validated method under GLP (Good Laboratory Practice). The laboratory will be unblinded and will conduct the analyses after the clinical study is completed but the dataset is still unlocked, ensuring that the sponsor remains blinded by recoding the subjects’ numbers. The three drugs will be identified and quantified using the HPLC method with tandem mass spectrometric detection (LC-MS/MS). For each compound, relevant PK parameters will be calculated by standard non-compartmental methods for those subjects with sufficient plasma concentration data using Phoenix 64® WinNonLin® (Version 6.3 or later) with a log-linear terminal phase assumption [2]. All reported sampling time deviations will be taken into consideration for evaluation of PK parameters.

As described for RCT 3 [2], for all three drugs, whenever applicable, the following standard non-compartmental pharmacokinetic parameters and drug exposure-related metrics will be estimated in each subject, including

- *C*_max_: maximum plasma concentration.
- *t*_max_: time to reach maximum plasma concentration.
- λz: the terminal phase constant will be calculated by linear regression of the last phase of the curve (log concentration vs. time).
- *t*_1/2_: terminal half-life will be determined with the expression *t*_1/2_ = 0.693/λz.
- AUC0-t: area under the plasma concentration-time curve from time zero to last quantifiable concentration calculated by the linear and/or log trapezoidal rule.
- AUC0-∞: The area under the curve of plasma levels vs. time from zero to infinite will be obtained with the expression AUC0-∞ = AUC0-t + Clast/λz, where Clast is the predicted plasma concentration at the last time measured.

A descriptive analysis will be provided for each derived PK parameter. Below limit of quantitation (BLQ) concentrations will be treated as zero for all statistical analyses.

Full details of the pharmacokinetic analysis and the corresponding statistical analysis of PK parameters will be described in the final report.

## Statistical methods

### Statistical methods for primary and secondary outcomes {20a}

**Fig. 4:**
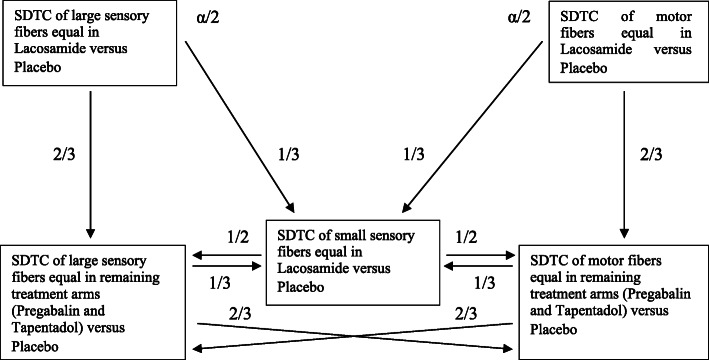
Statistical procedure. The figure depicts the sequentially rejective multiple testing approach used for the selected confirmatory analyses. The two primary endpoints will be tested in parallel, splitting the overall *α* equally between the endpoint tests. If any of these two tests shows significant differences, key secondary tests will be conducted using the α-levels as passed on from initial/prior tests according to specified weights

The statistical procedure for the selected, confirmatory analyses will follow the sequentially rejective multiple testing approach described by Bretz et al. [33]. The two primary endpoints, SDTC of large sensory fibers and SDTC of motor fibers, will be tested for their differences between the treatment arms lacosamide vs. placebo, first. Lacosamide is chosen because of expected activity on the peripheral nervous system. This will be conducted in parallel, splitting the overall α equally between the endpoints’ tests, i.e., each test has a type I error of α/2. If any of these two tests shows significant differences, key secondary analyses will be conducted using the α-levels as passed on from initial/prior tests according to specified weights. The exact procedure (with the local levels as well as the weights with which to pass α-levels on) is illustrated in Fig. 4.

### Primary analysis of endpoints

Large sensory fiber endpoint data being repeated measurements (at the first PD time point post dose) across the four periods will be analyzed with a mixed effect model with treatment (4 levels), period (4 levels) and center and sequence as fixed effects. The variance-covariance structure for the repeated measure variable period should be chosen as compound symmetry (CS). The least squares (LS) means difference of treatment lacosamide vs. placebo will be estimated and tested based on this model and the estimate as well as the corresponding unadjusted confidence interval and *p*-value will be provided. Test of motor fiber endpoint will be similar and specified in the statistical analysis plan. If either (or both) tests of the primary endpoints show significant differences between lacosamide and placebo in the sense that the *p*-value is below the corresponding local alpha level, the differences are confirmed and the testing procedure continues according to the figure above. The method for testing the primary endpoints comparing the other treatment groups vs. placebo mirrors the model specifications of the primary analysis above but using the Dunnett-adjusted estimation and testing results to compare pregabalin and tapentadol with placebo respectively. The method for testing the key secondary endpoint in small sensory fibers is identical to the specifications of the primary analysis above.

### Interim analyses {21b}

No interim analysis is foreseen for this study.

### Methods for additional analyses (e.g., subgroup analyses) {20b}

Several additional secondary analyses will be done, such as variants of primary endpoint parameter extraction, additional functional biomarkers extracted from the recorded signals, item analyses of PROMs, pharmacokinetic-pharmacodynamic modelling, complex hierarchical modelling, estimation of variance, and effect size of candidate endpoints for future clinical trials. Further details will be provided in the statistical analysis plan.

The data collected in this trial together with the data from the other three IMI2-PainCare-BioPain-RCTs and preclinical studies of the BioPain project will be subject to pharmacometric analyses with the intention to validate biomarkers that can translate from preclinical to clinical readouts [2]. The expected analyses will be described in a separate pharmacometric analysis plan and will consist briefly in developing population pharmacokinetic/pharmacodynamics (PK/PD) models estimating the primary PK parameters (i.e., apparent volume of distribution, and total plasma clearance), the primary PD parameters (i.e., C50, the plasma or effect site concentration that elicits a response equal to half of the maximum attainable effect (EMAX)), and their associated inter-individual variability [2]. Analyses of demographic data and other baseline characteristics will consist of descriptive summary statistics. Assessment of the repeatability of an individual biomarker response measurement method will be performed using the statistics described by Bland & Altman [34] (e.g., differences against mean plots) and as described previously [2].

### Methods in analysis to handle protocol non-adherence and any statistical methods to handle missing data {20c}

The analysis method of the mixed effect model already takes missing data into account and missing data issues are also partly addressed by the sensitivity analyses [2]. More thorough and complete descriptions of missing data handling will be provided in the statistical analysis plan.

### Plans to give access to the full protocol, participant level-data and statistical code {31c}

This will be determined at the end of the project period of IMI-PainCare.

## Oversight and monitoring

### Composition of the coordinating center and trial steering committee {5d}

This trial is part of the Biopain subtopic of the IMI2 PainCare project. As described for RCT3 [2], the PainCare Innovative Medicines Initiative (www.imi-paincare.eu) is a partnership between the European Union and the European pharmaceutical industry. The Project Coordinator is Prof. Rolf-Detlef Treede, Heidelberg University, Germany. The sponsor and leader of the RCT described in this paper is Prof. Nanna Brix Finnerup, Aarhus University, Denmark. There will be one Principal Investigator at each trial site. Trial-related duties and responsibilities will be specified in the site delegation list. As previously described [2], the IMI-PainCare project has an external ethics advisory board which (1) reviews the proper application of the relevant laws and guidelines containing ethical rules and the H2020 rules by the investigators; (2) provides advice to and monitors the activities of the investigators with regard to ethical issues; and (3) provides advice on the compliance with European ethical laws and regulations and with different guidelines, laws and regulations of countries, where studies will be performed. ConsulTech, a non-clinical partner of the study, will collect all relevant ethical documents and will ensure that all partners submit them on time. ConsulTech will then draw up a questionnaire for the ethics advisory board, which will allow it to check whether all important ethical requirements and documents have been submitted and that all legal guidelines have been adhered to.

### Composition of the data monitoring committee, its role and reporting structure {21a}

A data monitoring committee is not foreseen. Data management will be performed by the Heidelberg University Computing Centre. Documentation of the responsibilities and delegation thereof will be maintained in the trial master file [2]. All aspects of the data management process, including data validation and query management, medical coding, ECG data, and biomarker data, and data lock procedures are described in the data management plan [2].

### Adverse event reporting and harms {22}

Adverse events will be documented from the time of enrollment (i.e., the time the informed consent form is signed) up to the time of the last protocol scheduled contact. Subjects will be asked to report adverse events (open-ended questions) and will also have the opportunity to report these spontaneously. Investigators will report all serious adverse events to the sponsor as soon as possible and within 24 h. Handling of adverse events will follow GCP and applicable regulations as described previously [2]. They will be coded using MedDRA. All adverse events (number and severity) will be reported in the publication.

### Frequency and plans for auditing trial conduct {23}

The non-clinical partner ConsulTech will coordinate trial monitoring activities. As described previously, trial site monitoring as defined in GCP will be performed by authorized personnel of a subcontracted Contract Research Organization (CRO) at pre-defined intervals and according to a monitoring plan depending on the progress of the trial. Corrections, amendments, or clarifying statements resulting from monitoring visits will be made by the investigator where necessary. Appropriate checking against source documents will be done. The Principal Investigator, any investigator(s), the sponsor, or personnel at other establishments, will cooperate with any inspection of the documents, facilities, records, and other resources deemed appropriate by the inspecting authorities to be related to the trial and that may be located at the trial site, at the sponsor, or at other establishments.

### Plans for communicating important protocol amendments to relevant parties (e.g., trial participants, ethical committees) {25}

As described for RCT3 [2], any modifications to the protocol which may impact the conduct of the study, potentially benefit the subject or affect the subject safety, will require a formal amendment to the protocol. Such amendment will be approved by the IEC prior to implementation and notified to the health authorities in accordance with local regulations.

### Dissemination plans {31a}

As described for RCT3 [2], a final report integrating trial results will be prepared. The Principal Investigator will provide the competent authority/ies and relevant IEC(s) with a summary of the trial results in accordance with applicable regulatory requirements. The results of this trial will be publicly disclosed (EudraCT). The results (or parts thereof) of this trial will be published according to the Grant Agreement and Consortium Agreement of IMI-PainCare (grant No 777 500).

## Discussion

IMI2-PainCare-BioPain-RCT1 is one of four RCTs aiming at validating biomarkers of drug effects on nociceptive processing using the same trial design and IMPs. The design of IMI2-PainCare-BioPain-RCT3 on electrophysiological brain biomarkers has been published before; two companion manuscripts describe IMI2-PainCare-BioPain-RCT2 (spinal biomarkers) and IMI2-PainCare-BioPain-RCT4 (brain imaging biomarkers). By looking at biomarkers of nerve excitability in both large and small nerve fibers, IMI2-PainCare-BioPain-RCT1 aims to profile efficacy of the three model compounds (lacosamide, pregabalin, tapentadol) on mechanisms that may contribute to ectopic impulse generation in neuropathic pain patients and hence to their ongoing pain levels. According to the sequentially rejective multiple testing design, two primary endpoints will be tested simultaneously at alpha/2; these are pairwise comparisons of one medication vs. placebo for one post-medication time point and one readout variable that are most likely to be significant based on published literature (see power calculation). Three key secondary endpoints are assessed, given that alpha levels were passed successfully from one or both of the primary endpoints or from one or both of the other key secondary endpoints; these tests include the remaining two medications vs. placebo and a third readout variable. Other types of analysis such as two-way ANOVAs, as well as efficacy analyses on other readout variables may be done as exploratory and hence are not listed here. All efficacy analyses will be done on data pooled across participating centers; efficiency of this multi-center study approach in human volunteers will be assessed by comparing across-center vs. within-center variability of the various readout variables.

In addition to the analysis of the individual RCTs, biomarker results and pharmacokinetic and pharmacodynamics analyses will be compared across trials and to preclinical studies and will possibly try to establish a latent variable model of underlying mechanisms at peripheral, spinal, and brain levels. Pain ratings will be included as another readout variable, and questionnaires on psychological traits (e.g. catastrophizing, anxiety) will be analyzed as potential predictors of pain and analgesia. By identifying specific mechanisms within different compartments of the nociceptive system in healthy volunteers, these same quantitative neurophysiological biomarkers have the opportunity, if well validated against clinically meaningful outcomes in patients, to permit patient stratification and enrichment in later clinical trials as encouraged by the recent EMA/CHMP/970057/2011 Guideline. This will accelerate the development of novel analgesics in several ways: preclinical prediction will be improved by using translatable readouts across species; clinical Phase 1 trials will benefit from biomarkers of target engagement and from human surrogate models predictive of clinical efficacy; clinical Phase 2 and 3 studies will benefit from tools for patient stratification.

## Trial status

This manuscript is based on protocol version 3.0 dated 15/05/2019. Recruitment started in July 2020 and after two extensions due to the COVID-19 pandemic is expected to be completed April 2022. Recruitment of subjects was disrupted several times between March 2020 and January 2022.

## Abbreviations

AV: Atrioventricular
CA: Competent authority
*C*_max_: Peak concentration
CRF: Case report form
IEC: Independent Ethics Committee
ECG: Electrocardiography or electrocardiogram
EFPIA: European Federation of Pharmaceutical Industry Associations
EMA: European Medicines Agency
EU: European Union
GCP: Good Clinical Practice
ICH: International Conference on Harmonization
IMI: Innovative Medicines Initiative (of the EU)
IMP: Investigational medicinal product
K2-EDTA: Dipotassium ethylenediaminetetraacetic acid
*N*: Number (of subjects)
NET: Nerve excitability testing
PD: Pharmacodynamics
PI: Principal Investigator
PK: Pharmacokinetics
PK-PD: Pharmacokinetics-pharmacodynamics
PR: Interval of the ECG
PROM: Patient-Reported Outcome Measure
RCT: Randomized controlled trial
SD: Standard deviation
SmPC: Summary of Product Characteristics
*t*_1/2_: Half-life
*t*_max_: Time to reach peak concentration

## Footnotes

1 EudraCT 2019-000942-36 ; https://clinicaltrialsregister.eu/ctr-search/trial/2019-000942-36/DK

2 EudraCT 2019-000755-14; https://clinicaltrialsregister.eu/ctr-search/trial/2019-000755-14/IT

3 EudraCT 2019-001204-37 ; https://clinicaltrialsregister.eu/ctr-search/trial/2019-001204-37/BE

4 EudraCT 2019-000908-15 ; https://clinicaltrialsregister.eu/ctr-search/trial/2019-000908-15/DK
